# How to use the *MEROPS* database and website to help understand peptidase specificity

**DOI:** 10.1002/pro.3948

**Published:** 2020-10-03

**Authors:** Neil D. Rawlings, Alex Bateman

**Affiliations:** ^1^ European Molecular Biology Laboratory European Bioinformatics Institute (EMBL‐EBI) Hinxton, Cambridge UK

**Keywords:** binding site, enzyme classification, peptidase, proteolytic enzyme, substrate specificity

## Abstract

The *MEROPS* website (https://www.ebi.ac.uk/merops) and database was established in 1996 to present the classification and nomenclature of proteolytic enzymes. This was expanded to include a classification of protein inhibitors of proteolytic enzymes in 2004. Each peptidase or inhibitor is assigned to a distinct identifier, based on its biochemical and biological properties, and homologous sequences are assembled into a family. Families in which the proteins share similar tertiary structures are assembled into a clan. The *MEROPS* classification is thus a hierarchy with at least three levels (protein‐species, family, and clan) showing the evolutionary relationship. Several other data collections have been assembled, which are accessed from all levels in the hierarchy. These include, sequence homologs, selective bibliographies, substrate cleavage sites, peptidase–inhibitor interactions, alignments, and phylogenetic trees. The substrate cleavage collection has been assembled from the literature and includes physiological, pathological, and nonphysiological cleavages in proteins, peptides, and synthetic substrates. In this article, we make recommendations about how best to analyze these data and show analyses to indicate peptidase binding site preferences and exclusions. We also identify peptidases where co‐operative binding occurs between adjacent binding sites.

## 1 OVERVIEW

The *MEROPS* website (www.ebi.ac.uk/merops) began in 1996 as a vehicle in which to present the classification of proteolytic enzymes into evolutionarily related clans and families. A proteolytic enzyme cleaves the peptide bond between two amino acids in a peptide or protein, and like any enzyme that degrades a biological polymer, had been difficult to classify by specificity alone. The specificity of a proteolytic enzyme can be complex, and many enzymes can show similar if not identical specificity but act in different environments or under different environmental conditions. The classification by Rawlings and Barrett^1^ was based entirely on sequence and structural relationships. Proteolytic enzymes with similar sequences were assembled into a family, and enzymes from different families were assembled into a clan if the structures were known (or thought to be) related. When no tertiary structure is known, it is usually not possible to assign a family to a clan. In some cases, if the order of the catalytic residues is the same in the sequence, then a family where the structure is not known can be provisionally assigned to an existing clan.

Many proteolytic enzymes are multidomain proteins with the proteolytic activity restricted to one structural domain. Only the sequence and structure of this single domain are taken into consideration when assigning a proteolytic enzyme into a family and clan.

The vary majority of proteolytic enzymes are peptidases (also known as proteases or proteinases) which cleave peptide bonds by hydrolysis (and form subclass 3.4 in the NC‐IUBMB Enzyme Nomenclature^2^). Peptidases vary in the nature of the nucleophile in the hydrolytic reaction, which can be the hydroxyl of a serine (“serine peptidase”), the hydroxyl of a threonine (“threonine peptidase”), the thiol of a cysteine (“cysteine peptidase”), water bound to aspartic acid residues (“aspartic peptidases”), water bound to glutamic acid residues (“glutamic peptidases”), or water bound to a metal ion (“metallopeptidases”). Within a family, almost all peptidases will be restricted to one catalytic type, and each family is given an identifier consisting of a letter to indicate the catalytic type (S, T, C, A, G, or M) followed by a number. A clan identifier consists of a letter to indicate the catalytic type followed by a second letter assigned sequentially. However, within a clan, catalytic type can vary, so additionally for a clan with mixed catalytic type the identifier begins with the letter P. For a family where the catalytic type is unknown, the identifier begins with the letter U. Asparagine lyases, which cleave themselves by rearrangement of an Asn to form a succinimide, are the only nonhydrolytic proteolytic enzymes, and for these the clan and family names begin with the letter N.^3^

Some families have been divided into subfamilies where there is strong evidence of a deep division within the family (e.g., when a sequence relationship was discovered that enabled two former families to merge, with each former family being retained as a subfamily within the new family). We wish to stress that in the *MEROPS* classification system, a subfamily is a major division within a family, but it is optional. Of the 275 families of proteolytic enzymes, only 41 are divided into subfamilies. Most families have only two subfamilies; the largest numbers are in families A2 (where there are six), C3 (eight), M28 (six), and S1 (six).

Similarly, some clans have been divided into subclans, where there is evidence that the catalytic mechanism differs between the families. Only five of the 47 clans are divided into subclans: Clan MA is divided into the Glu‐zincins, Asp‐zincins, and Met‐zincins; Clans PA, PB, PC, and PD, all of which are of mixed catalytic type, are divided into individual subclans of either cysteine, serine, or threonine peptidases.

A third level in the hierarchy was introduced in 1998, which we have termed a “protein‐species,” which represents the same enzyme from different organisms.^4^ Each protein‐species is given a unique MEROPS identifier consisting of the family name (padded with a zero to be at least three characters) followed by a dot and a sequential number. With only a few exceptions for model organisms with completely sequenced genomes, a *MEROPS* identifier is only established for a biochemically characterized protein. In any family, there will be homologs that cannot be assigned to a *MEROPS* identifier. It is only at the level of protein‐species that specificity is taken into account. Unfortunately, some other databases that also classify protein sequences have used the term “subfamily” to represent the protein‐species level, which has caused confusion.

Some peptidases function in a complex of proteins. Where this complex contains more than one peptidase, a special *MEROPS* identifier is used. If all peptidases in the complex are homologous, then the special identifier consists of an “X,” followed by the family identifier, a dot, and a sequential number. For example, the eukaryotic 20S proteasome is XT01.001. For a protein containing more than one peptidase unit, a similar special identifier is used, for example, metallocarboxypeptidase D is XM14.001. Where the complex contains peptidases from different families, the letter “P” followed by a number is used instead of the family name, for example, the tricorn peptidase complex is XP01.001. Each characterized, individual peptidase (or peptidase unit) from a complex is also given a normal *MEROPS* identifier. Special identifiers are not created for viral polyproteins because an individual enzyme is functional only on separation from the polyprotein.

There are proteins with no hydrolytic activity but which are related to peptidases. This means that within any family of proteins, function is not necessarily conserved. Often loss of activity is associated with loss or replacement of catalytic residues, enabling an uncharacterized protein to be identified as a nonpeptidase homolog from its sequence. Within some families of peptidases, some homologs have different enzymatic activities, for example, family S9 includes lipases and esterases. For a characterized nonpeptidase homolog, a special *MEROPS* identifier is established in which the first number after the dot is a nine.

There are many biochemically characterized peptidases for which a sequence is not known, or only a short fragment is known which cannot be assigned to a family. Special identifiers are created for these, using a letter to represent the catalytic type followed by a “9,” and a letter to indicate the kind of peptidase activity: A for an aminopeptidase, B for a dipeptidase, C for a dipeptidyl‐peptidase, D for a peptidyl‐dipeptidase, E for a carboxypeptidase, and G for an endopeptidase. For example, membrane Pro‐Xaa carboxypeptidase is M9E.004.

Thus in the *MEROPS* classification there is a multilevel hierarchy, from sequence to protein‐species, to subfamily (if any), family, subclan (if any) and clan.

**FIGURE 1:**
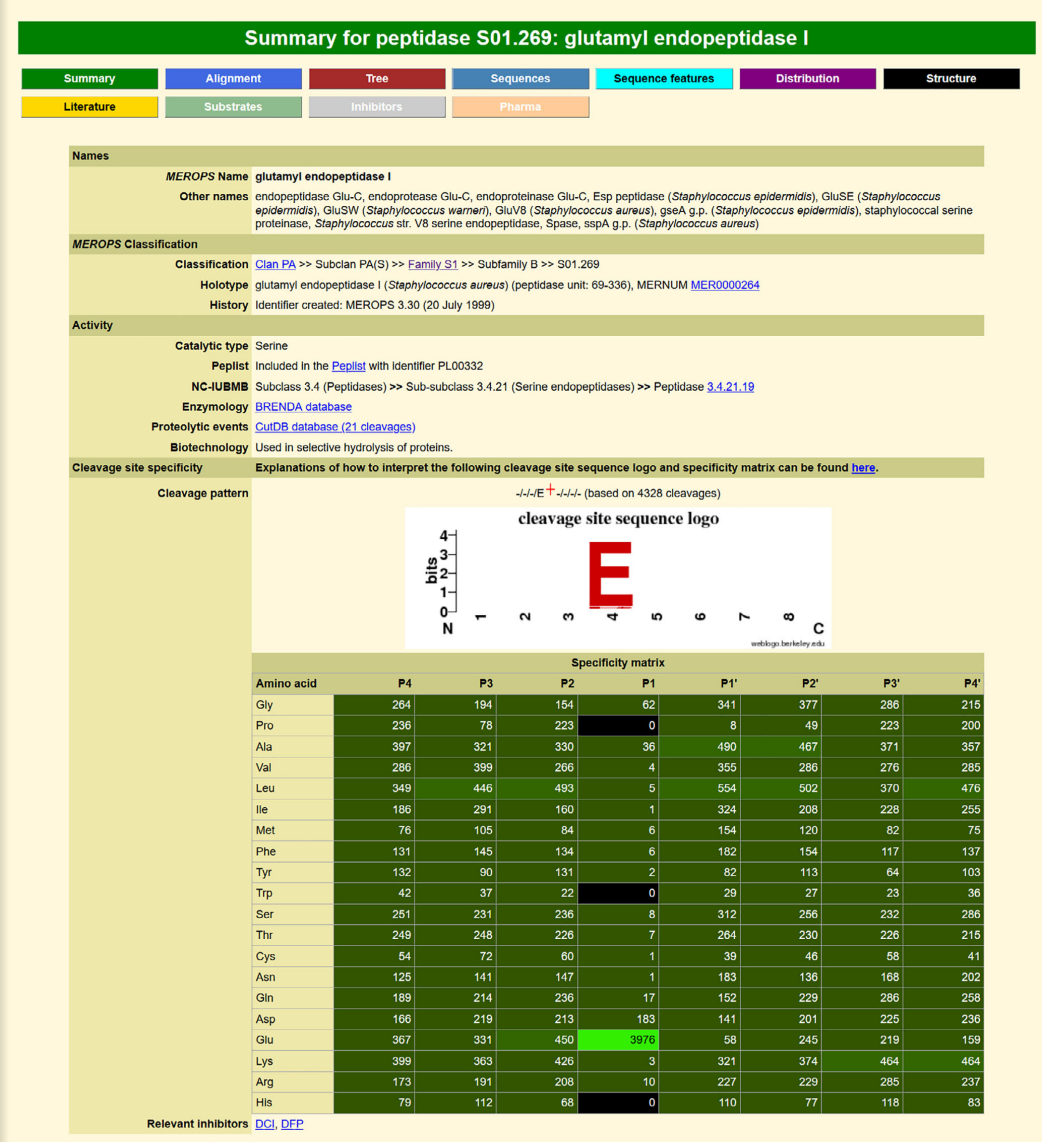
An example peptidase summary page from the *MEROPS* website. The peptidase summary page for glutamyl endopeptidase I is shown

Once the *MEROPS* hierarchy was established, it was possible to expand the website to include other items that could be linked to a level in the hierarchy. The first of these was an extensive bibliography which could be linked at protein‐species, family, and clan levels. The bibliography is updated fortnightly. Sequence alignments and phylogenetic trees were added at the family level^5^; these are usually regenerated for each release. A collection of known cleavage sites in proteins was established from the scientific literature which could be linked at the protein‐species level.^6^, ^7^ Peptidase–inhibitor interactions were included at the protein‐species level.^8^ Both of these collections are updated periodically. A summary page is presented on the website for each holotype, family, and clan. An example peptidase summary page is shown in Figure 1. Buttons across the top of the screen provide access to supplementary pages, which for a peptidase summary page includes substrate cleavages, inhibitor interactions, and a bibliography. Tables on the summary page show alternative names, the full *MEROPS* classification, and details of activity. Substrate specificity is shown as a logo and as a matrix showing the amino acids occupying binding pockets P4‐P4'. Both are calculated dynamically from all the known substrate cleavages for this enzyme. In the matrix, different shades of green highlighting are used to indicate preference, with the brighter green showing most restricted specificity. Finally, there is a list of important inhibitors, with links to the relevant inhibitor summary pages.

The methodology used to classify proteolytic enzymes was extended to include peptidase inhibitors that are proteins.^9^ An identifier for a family of inhibitors begins with the letter I. In most cases, an inhibitor family has a unique tertiary structure, so in most cases a clan contains only one family. Because there are 34 clans of inhibitors, identifiers starting with more than one letter were required, and the identifier for a clan of inhibitors begins either with an I or a J. For compound inhibitors, similar special *MEROPS* identifiers were also created, but beginning with the letter “L”; for example, LI01.001 for ovomucoid.

Small molecule inhibitors (SMIs) were added to the *MEROPS* website in 2008.^8^ These are not classified and are simply listed alphabetically.

All the features available on the *MEROPS* website were detailed in a recent publication.^10^ Few new features have been added, but the data underlying these features are kept up to date. The most recent release (release 12.2) of the MEROPS database was June 2020. In this report, we will discuss the classification and the substrate cleavage collection, in particular how to use the data to predict the specificity of proteolytic enzymes and present some analyses of that data.

## 2 METHODS

### 2.1 *Type examples*

Our classification of proteolytic enzymes and inhibitors has been influenced by organism taxonomy. When an organism is described for the first time, the identification of a type specimen is the first step. The equivalent for a protein would be the source organism and sequence from the publication where it was first characterized biochemically. This sequence is known as the *holotype*. In the *MEROPS* classification, there is a holotype for each protein‐species. One of these holotypes will be selected as the *type example* for a family (or subfamily): this is usually the first protein in the family (or subfamily) to be biochemically characterized. For a clan, there is a *type structure*, which is usually the first solved tertiary structure for a protein from any of the families included in the clan. Only that part of the sequence which includes the catalytic machinery (the active site residues and the primary substrate‐binding sites but not a secondary binding site such as an *exosite*) are considered for a proteolytic enzyme, and only that part of the sequence which includes the reactive site are considered for an inhibitor. We term these parts of the sequence the *peptidase unit* and the *inhibitor unit*, respectively. When active or reactive site residues are not known, the peptidase or inhibitor unit is estimated, omitting parts of the sequence known to have other functional domains and targeting signals as identified by Pfam.^11^

A new holotype and *MEROPS* identifier is set up when a protein is characterized and either has a different specificity to any other protein in the family, or the specificity is similar, but the protein acts in a different cellular location, has a different architecture, or the sequence does not cluster in a phylogenetic tree with that of the existing holotype(s) that have similar specificity.

The same *MEROPS* identifier is assigned to uncharacterized homolog of the holotype that are considered to represent the same protein from different species. Our criteria for doing this were set out^12^ and include more than 50% sequence identity within the peptidase/inhibitor unit, preservation of the order and number of domains, similar targeting signals and transmembrane regions, and the homolog and the holotype cluster together on a phylogenetic tree.

### 2.2 *Assembly of families*

Homologs are assigned to a family from a BlastP^13^ or HMMER^14^ search using the peptidase/inhibitor unit sequence from an existing member of the family (usually that of the holotype). Homologs are collected from either the nonredundant Protein sequence library at NCBI^15^ or UniProt.^16^ All homologs returned from the search with an E‐value less than 0.001 are assigned to the family. There are instances when a search with a sequence from one family will return homologs already assigned to another family; if all the active site residues are aligned, then the families will be merged (with each former forming a subfamily in the new family). If not all active site residues align, then the families are not merged, and this frequently happens among families within clans SC and MA.

Protein sequence alignments are made with *Muscle* ^17^ and phylogenetic trees using *QuickTree*.^18^

### 2.3 *Assembly of clans*

Co‐ordinates for a peptidase (or inhibitor) structure are downloaded from the Protein DataBank (PDB)^19^ and submitted to the Dali server.^20^ Any structures returned showing similarity at 6 *SD* units or more are considered to be homologous, and all of those that are peptidases (or peptidase inhibitors) with be included in the same clan, provided the similarity covers the peptidase (or inhibitor) units.

### 2.4 *Substrate cleavage collection*

Known cleavage sites in substrates are collected from the literature and include cleavages in proteins, peptides, and synthetic substrates. Cleavages that are physiological, pathological, or nonphysiological are collected to maximize the number for each peptidase. For proteins and peptides, cleavages are mapped to their respective UniProt entries. The cleavage position (the scissile bond) is recorded as the position of the P1 residue according to the residue numbering in the UniProt entry. Four residues either side of the scissile bond are recorded, representing residues P4‐P4' (according to the binding site nomenclature of Schechter and Berger^21^).

### 2.5 *Peptidase specificity analysis*

A nonredundant set of cleavages for each peptidase was made by replacing all nonstandard amino acids and synthetic blocking groups with the letter “X” and then selecting distinct P4‐P4' sequences. For further analysis, only those peptidases with 20 or more nonredundant cleavages were considered. The frequency of each amino acid for each peptidase binding site could then be counting, and binding sites occupied by only one or two amino acids could be identified. By converting each amino acid to one of six types, a binding site occupied by one type or not occupied by one type could also be identified. The types were acidic (Asp, Glu), basic (Arg, Lys, His), aliphatic (Ile, Leu, Val), aromatic (Phe, Tyr, Trp), small (Ala, Gly, Ser), and other (Asn, Cys, Gln, Met, Pro, Thr). Binding sites where one or two amino acids (or one amino acid type) were never found could also be identified, but to exclude the rarity of amino acids such as tryptophan affecting the results, the number of cleavages per peptidase was increased to 30 or more. To account for instances where the substrate cleavage data collected from the literature might be in error, substrate‐binding sites occupied by one amino acid or one amino acid type in more than 90% of the cleavages were calculated for each peptidase.

Examples of co‐operative binding, where the binding of an amino acid in one site affects what can bind in a neighboring site, were also searched for. For example, Phe only binds in P1 if Gly binds in P2. Because the frequency of occurrence of an amino acid will affect how often it is found with another amino acid in the P4‐P4' sequence, there had to be 10 or more instances where two amino acids occurred together, and to exclude the preferences previously observed, each amino acid had to occur in less than 90% of the cleavages for the peptidase in question.

## 3 RESULTS

Table 1 shows the number of *MEROPS* identifiers, families and clans for proteolytic enzymes and inhibitors.

**TABLE 1:** Counts of identifiers, families and clans in the *MEROPS* database

	Proteolytic enzymes	Protein inhibitors
*MEROPS* identifiers	4,684	734
Families	276	83
Clans	47	34

Numbers of other features included on the website are shown in Table 2.

**TABLE 2:** Counts of other features in the *MEROPS* database

Item	Total
References	70,628
Substrate cleavages	98,378
Peptidase–inhibitor interactions	6,854
Small‐molecule inhibitors	1,345

Of the 4,684 *MEROPS* identifiers, 1,424 are assigned to holotypes that are uncharacterized peptidase homologs from model organisms. The remaining 3,260 holotypes are known to be active as peptidases, but cleavage sites in substrates are only known for 1,342 different peptidases (27.6% of all holotypes, but 41.2% of all holotypes known to be active peptidases). In addition to the number of cleavages shown above, a further 4,173 cannot be assigned to a single peptidase. For example, some 3,694 eukaryotic protein precursors are known to have the initiating methionine (Met1) removed by either methionyl aminopeptidase 1 (M24.001) or methionyl aminopeptidase 2 (M24.002), but because it is not known which, the cleavages are assigned to an “M24 homolog.” The number of cleavages that can be mapped to UniProt entries is 94,805 of which 32,472 are physiological or pathological. There are 6,300 cleavages in synthetic substrates. The peptidase with most known cleavages is trypsin 1 (S01.151) with 22,528; this is because trypsin 1 is widely used in proteomics studies to degrade whole proteomes prior to mass spectroscopy. The peptidase with most physiological or pathological cleavages is matrix metallopeptidase‐3 (M10.005) with 2,452.

Table 3 shows the frequency that amino acids occur in all non‐redundant cleavages sites, in terms of percentage. Amino acids that occur in more than 10 % of cleavages are highlighted in yellow. These are Leu in P2, Arg and Lys in P1, and Ala and Leu in P1'. Amino acids that occur in less than 1 % of cleavages are highlighted in orange. These are Trp in any position, and Cys and Ile in P1. Both Trp and Cys are the least frequent amino acids, and Cys may occur in a disulfide bond, which probably prevents cleavage. The low frequency of Ile in P1 is unexpected.

**TABLE 3:** Percentages of amino acids in substrate‐binding sites P4‐P4'

Amino acid	P4	P3	P2	P1	P1'	P2'	P3'	P4'
‐	10.5	8.2	6.5	0	0	4.3	5.3	6.2
Ala	7.6	9	8.5	7	11.1	8.9	7.9	7.3
Arg	4.6	4	3.7	17	3.1	4.1	3.3	3
Asn	2.9	2.8	2.8	4	2.9	3	3.5	3.5
Asp	4.9	3.8	2.7	8.5	4.4	4.6	5.6	7.1
Cys	1.2	1.3	1.2	0.9	1.1	1	1.3	1.1
Glu	6.5	6.9	6	8.2	4.9	6.7	7.6	8.7
Gln	3.9	4.1	4.1	2.8	3.1	4.4	4.5	4.5
Gly	6.7	6.5	6.3	5.8	7.8	6.9	8.3	7.6
His	1.9	1.8	1.6	1.2	1.8	2	2	1.9
Ile	4.4	4.7	4.6	0.7	5.7	5.2	4.4	4.2
Leu	7.8	8.4	11.7	5.6	11	8.9	7.9	6.9
Lys	5.2	5.1	5	15.7	6.8	5.2	5.1	5
Met	1.9	2	2	6	2.4	1.8	1.7	1.5
Phe	3.1	3.5	4.3	3.8	4.5	3.4	3.2	2.9
Pro	5.8	5.6	5.6	2	1.5	5.2	6.2	7.3
Ser	6	6	5.9	3.6	9.3	6.8	6.8	6.5
Thr	4.4	4.7	4.4	2.6	4.7	5.6	5.2	5.3
Trp	0.8	0.7	0.9	0.7	0.7	0.8	0.8	0.8
Tyr	2.3	2.2	2.7	2.5	2.8	2.7	2.4	2.1
Val	6.5	7.6	8.6	1.4	7.2	8	6.6	6.1
X	1	0.9	0.9	0.2	3	0.3	0.3	0.4

*Note*: Standard amino acids are included, plus “‐” to indicate an unoccupied site and “X” to indicate a nonstandard amino acid or other moiety. An amino acid that occupies a substrate‐binding site in 10% or more cleavages is highlighted in yellow. An amino acid that occupies a substrate‐binding site in less than 1% of cleavages is highlighted in orange.

The number of holotypes for which 20 or more nonredundant cleavages are known is only 217 (4.6%), and it is these for which substrate preferences are analyzed below.

Peptidases where only one or two amino acids, or one amino acid type, are accepted in P4‐P4' are shown in Figure S2. This figure also shows binding sites where one or two amino acids (or a single amino acid group) are unacceptable. Preferences for 168 peptidases are shown, representing 77% of peptidases with 20 or more known substrate cleavages. For the remaining 49 peptidases, the specificity is cryptic and cannot be explained in such simple terms. Peptidases not in Figure S2 include cathepsin L (C01.032; 2,862 cleavages), matrix metallopeptidase‐2 (M10.003; 2,558), matrix metallopeptidase‐3 (M10.005; 2,425), meprin alpha subunit (M12.002; 771), meprin beta subunit (M12.004; 925), granzyme B (*Homo sapiens*‐type, S01.010; 1,636), and the 20S constitutive proteasome peptidase complex (eukaryote, XT01.001; 675). Granzyme B apparently cleaves after residues other than Asp and Glu.^22^, ^23^

There is a common misconception that only the P1 binding pocket of a peptidase is important, perhaps because peptidases from the well‐studied families C14, S1 and S8 all show such limited specificity. Figure S2 clearly shows a preference in any of the binding pockets P4‐P4' for some peptidases. The P1 pocket, however, shows most instances of limited preference (in 61 peptidases), whereas fewest peptidases show preference in P4 (seven peptidases) or P4' (six). However, when looking at unacceptable binding, the P1 pocket shows least (30), whereas the other binding pockets show unacceptable residues in between 34 and 48 peptidases.

All peptidases have to accept at least one residue in P1 and P1'. For a dipeptidase, there are no other binding pockets, for an aminopeptidase P4‐P2 do not exist, and for a carboxypeptidase P2'–P4' do not exist. A peptidase that releases a dipeptide from the N‐terminus of a peptide (a “dipeptidyl‐peptidase”) does not have P4 or P3, and a peptidase that releases a dipeptide from the C‐terminus of a peptide (a “peptidyl‐dipeptidase”) does not have P3' and P4'. In Figure S2, there are apparently 15 aminopeptidases, nine carboxypeptidases, four dipeptidyl‐peptidases, one peptidyl‐dipeptidase, and two peptidases that act like peptidyl‐tripeptidases.

Clearly, there are some anomalies in Figure S2. The DmpA peptidase from *Ochrobactrum anthropi* (*MEROPS* identifier P01.001) has been shown to be an aminopeptidase, but it is also thought to be self‐processing (and then acts as an endopeptidase),^24^ hence it does not show the characteristics of an aminopeptidase in Figure S2. Carboxypeptidase Q (M28.014) appears to be a dipeptidase in Figure S2, which reflects its former characterization as a lysosomal dipeptidase.^25^ SplE peptidase (S01.312) from *Staphylococcus aureus* was characterized by use of a cellular library of peptide substrates (CLiPS), in which residues beyond P1' were not identified,^26^ hence it appears in Figure S2 as if it were a carboxypeptidase. Sedolisin‐B (S53.002) and kumamolisin‐B (S53.005) were characterized using synthetic chromogenic substrates that covered P4‐P3' only,^27^ given the false impression of a peptidyl‐tripeptidase (an activity not known to exist).

The commonest preference shown in Figure S2 is that of either Arg or Lys (or “basic”) in P1, which occurs in 36 peptidases (but 20 of these are peptidases from family S1 and nine from S8). A preference for Arg or Lys occurs in P1' for CPG70 carboxypeptidase from *Porphyromonas gingivalis* (M14.023). Preferences for Ala, Asp, Gly, Met, and Val each occur in substrate sites from three peptidases. On the other hand, there is no preference for His or Trp in any of the binding sites shown in Figure S2, and Cys and Ile are each observed on only one occasion.

The residue most frequently excluded from a substrate‐binding site is Trp, which is not found in at least 45 of the sites shown in Figure S2, plus many others in combination with another amino acid (Cys and Trp are excluded from 15 binding sites, and Met or Trp from six). In addition, Cys is absent from at least 49 binding sites. Both Trp and Cys are the amino acids, which occur with the lowest frequency. However, evidence that these are real exclusions and not just the result of a low level of occurrence is that the number of binding sites in which Trp is not found is the same if in the analysis the number of cleavages per peptidase is increased to 50 or reduced to 10. It is also noteworthy that Ile is excluded from more sites than Pro, His, or Met, and that acidic residues are excluded from 11 sites.

The binding of a substrate to an enzyme is often described as a “lock and key” hypothesis, but this is misleading, because it implies a rigidity that is not present in either substrate or the enzyme. Peptidases are no exception, and there is a degree of plasticity in the active site. For example, if a large amino acid binds in one pocket, then this may prevent anything other than a small amino acid binding in the adjacent pocket. This is known as co‐operative binding, and 37 examples from 23 proteolytic enzymes have been identified and are shown in Table 4.

**TABLE 4:** Co‐operative binding in substrate‐binding sites of proteolytic enzymes

*MEROPS* identifier	Recommended name	Cleavages	Frequency	Site	Residue n	Residue n + 1
A01.002	pepsin B	25	12	P2	Gly	Phe
A01.002	pepsin B	25	17	P2'	Arg	Leu
A01.014	candidapepsin SAP1	25	15	P4	Leu	Val
A01.014	candidapepsin SAP1	25	14	P2'	Ala	Glu
A01.060	candidapepsin SAP2	21	15	P4	Leu	Val
A01.060	candidapepsin SAP2	21	15	P3	Val	Glu
A01.060	candidapepsin SAP2	21	15	P2'	Tyr	Leu
A01.060	candidapepsin SAP2	21	15	P3'	Leu	Val
A02.002	HIV‐2 retropepsin	26	10	P1'	Pro	Ile
A02.063	walleye dermal sarcoma virus retropepsin	23	10	P3'	Val	Gln
C01.100	cruzipain 2	26	16	P3'	Lys	Gln
C14.001	caspase‐1	171	18	P2	Pro	Asp
C14.001	caspase‐1	171	15	P2	Thr	Asp
C14.004	caspase‐7	466	13	P2	Phe	Asp
C14.004	caspase‐7	466	14	P2	Met	Asp
C30.005	SARS coronavirus picornain 3C‐like peptidase	24	13	P2	Gly	Gly
G01.001	scytalidoglutamic peptidase	35	28	P3	Lys	Leu
G01.001	scytalidoglutamic peptidase	35	16	P1'	Ser	Ser
G01.001	scytalidoglutamic peptidase	35	28	P2'	Ser	Lys
M01.018	endoplasmic reticulum aminopeptidase 1	47	13	P3'	Asn	Lys
M03.002	neurolysin	118	14	P2	Gly	Phe
M03.011	tropolysin	31	19	P3	Phe	Ser
N10.002	intein‐containing replicative DNA helicase precursor	80	42	P2	His	Asn
N10.002	intein‐containing replicative DNA helicase precursor	80	10	P1	Pro	Cys
N10.004	intein‐containing translation initiation factor IF‐2 precursor	30	15	P2	His	Asn
N10.004	intein‐containing translation initiation factor IF‐2 precursor	30	11	P3	Val	His
S01.136	granzyme B, rodent‐type	360	20	P2	Gln	Asp
S01.217	thrombin	163	16	P2	Ala	Arg
S01.217	thrombin	163	18	P2	Gly	Arg
S01.251	kallikrein‐related peptidase 4	116	21	P2	Lys	Arg
S01.251	kallikrein‐related peptidase 4	116	12	P2	Arg	Arg
S01.267	streptogrisin E	23	14	P1'	Val	Phe
S08.070	kexin	184	13	P3	Lys	Lys
S09.010	oligopeptidase B	35	10	P2	Arg	Arg
S50.004	blotched snakehead birnavirus Vp4 peptidase	30	12	P2	Ala	Ala
S50.004	blotched snakehead birnavirus Vp4 peptidase	30	11	P1'	Glu	Ala
S53.011	scytalidolisin	20	12	P1'	Ala	Ala

No co‐operative binding labeled P4' is shown, because the next residue would be P5' and only residues occupying P4‐P4' are in the *MEROPS* collection. The site showing co‐operative binding among most proteolytic enzymes in P2 (16 enzymes). In some cases, this reflects the perceived specificity at P1, rather than co‐operative binding. For example, caspase‐1 is thought to cleave only aspartyl bonds, yet other cleavages have been observed in a proteomics study^28^: of the nonredundant cleavages for caspase‐1, only 83% are of aspartyl bonds.

Amino acids most frequently involved in co‐operative binding are Arg, Ala, Lys, and Val. Trp is not observed at all, and Cys, Met, Thr, and Tyr occur just once.

## 4 DISCUSSION

All the tables from the *MEROPS* MySQL database can be downloaded freely from the *MEROPS* FTP site (ftp://ftp.ebi.ac.uk/pub/databases/merops/current_release/database_files), including the tables that form the *MEROPS* substrate cleavage collection (Substrate_search and cleavage). Tables are available in SQL format and as comma‐delimited text files.

Users of the *MEROPS* substrate cleavage collection who wish to analyze the data are reminded that the data set is not nonredundant, and it is recommended that a nonredundant subset is generated from it. The collection includes cleavages of synthetic substrates, as well as physiological and nonphysiological cleavages in peptides and proteins, and further subsets should be made as required, for example. to analyze only cleavages in physiological substrates.

There has been a tendency when analyzing peptidase preference to concentrate only on the substrate‐binding sites adjacent to the cleavage site, namely P1 and P1', and although for many peptidases specificity is directed to P1, this is only the case with peptidases from a minority of families. It is as important to identify which amino acids do not bind to a subsite as it is to identify those that do, and we have shown particular amino acids are more likely to be excluded from subsites other than P1. The substrate‐binding site of a proteolytic enzyme has some degree of plasticity, which we have shown from the identification of adjacent subsites where co‐operative binding may take place.

Much of the data in the *MEROPS* substrate cleavage collection has come from high‐throughput proteomics studies. In such studies, hundreds of cleavages are identified, the vast majority of which conform to the known specificity of the peptidase being studied. However, cleavages after amino acids thought not to correspond to the specificity of the peptidase have also been observed. Peptides are usually generated from an entire proteome by two proteolytic cleavages, one by a peptidase with known, limited specificity (such as trypsin) and the other by the peptidase under study (the “test peptidase”). Because the sequences of all the proteins in the proteome are known, and cleavages by the peptidase with limited specificity can be calculated, cleavages by the test peptidase can also be calculated from the mass of each peptide. Performing the same digestion only by the peptidase with limited specificity provides the control. Two cleavages are required to generate short peptides, because this reduces the number of candidate source proteins and the computing time required.^29^

As can be seen from Figure 1, there are many cleavages identified for glutamyl endopeptidase I which do not correspond to its “known” specificity: preference for Glu (92% of cleavages) or Asp (4%) in P1. Given that glutamyl endopeptidase I is regularly used as a peptidase with known limited specificity in these proteomics experiments, cleavages other than at glutamyl and aspartyl bonds could potentially lead to erroneous assumptions about which peptides are generated by the test peptidase when a proteome is digested. It is possible that under proteomics conditions, nonpreferential cleavages occur, resulting in these unusual cleavages, but it is also possible that these unusual cleavages are either a result of another, contaminating peptidase, or that the peptide has been mapped to an incorrect protein, perhaps because a splice variant has been misidentified in the proteome.

## AUTHOR CONTRIBUTIONS

**Neil D. Rawlings:** Conceptualization; data curation; methodology; software; visualization; writing‐original draft. **Alex Bateman:** Funding acquisition; project administration; resources; supervision; writing‐review and editing.

## Supporting information


**Supplementary Figure 2** **Identified preferences in binding sites P4‐P4’ for all peptidases.**
The figure shows each binding site where only one or two amino acids (or one amino acid type) are accepted, or in bold text where an amino acid (or amino acid type) is accepted in 90% or more of cleavages known for a peptidase. Where one or two amino acids (or one amino acid group) is not acceptable in a binding site, the cell is highlighted in black and the text is in white. Where a non‐standard amino acid or an N‐ or C‐terminal blocking group is the apparent preference, the cell is highlighted in yellow. Rows are arranged in alphabetical order of the *MEROPS* identifier (except when this is a peptidase complex, in which case the row follows other peptidases in the same family). The recommended name of the peptidase (or peptidase complex) is given, as is the number of known cleavages.
