# Supplementary material for: How to use the MEROPS database and website to help understand peptidase specificity
Source: Protein Sci. 2020 Oct 3;30(1):83–92. doi: 10.1002/pro.3948 (PMC7737757; doi:10.1002/pro.3948)
Supplement: Supplementary file 1 — Supplementary Figure 2 Identified preferences in binding sites P4‐P4’ for all peptidases. The figure shows each binding site where only one or two amino acids (or one amino acid type) are accepted, or in bold text where an amino acid (or amino acid type) is accepted in 90% or more of cleavages known for a peptidase. Where one or two amino acids (or one amino acid group) is not acceptable in a binding site, the cell is highlighted in black and the text is in white. Where a non‐standard amino acid or an N‐ or C‐terminal blocking group is the apparent preference, the cell is highlighted in yellow. Rows are arranged in alphabetical order of the MEROPS identifier (except when this is a peptidase complex, in which case the row follows other peptidases in the same family). The recommended name of the peptidase (or peptidase complex) is given, as is the number of known cleavages. [file PRO-30-83-s001.docx]

| *MEROPS* identifier | Recommended name | Cleavages | P4 | P3 | P2 | P1 | P1' | P2' | P3' | P4' |
| --- | --- | --- | --- | --- | --- | --- | --- | --- | --- | --- |
| A01.001 | pepsin A | 414 |  |  |  | Cys |  |  |  |  |
| A01.003 | gastricsin | 49 |  |  |  |  |  |  | Arg/Asn |  |
| A01.009 | cathepsin D | 758 |  |  |  | His | His | Trp |  |  |
| A01.010 | cathepsin E | 1356 |  |  |  | Pro | His |  | His |  |
| A01.012 | rhizopuspepsin | 248 |  | Cys |  | Cys | Cys |  |  |  |
| A01.016 | aspergillopepsin I | 156 |  | Cys | Cys | Cys | Cys | Cys/Trp |  |  |
| A01.020 | phytepsin | 52 |  |  |  |  |  | Phe/Trp |  |  |
| A01.053 | necepsin-1 | 122 | Ile/Trp | Ile/Trp |  | Ile/Met | Met |  | Cys | Ile/Met |
| A01.061 | candidapepsin SAP3 | 20 | Asn/**Leu** | **Val** | **Glu**/Lys |  |  | Thr/**Tyr** | **Leu**/Val | Pro/**Val** |
| A01.064 | candidapepsin SAP6 | 20 | **Leu** | **Val** | **Glu** |  |  | **Tyr** | **Leu** | **Val** |
| A02.001 | HIV-1 retropepsin | 850 |  |  | Lys/Pro | Lys/Thr |  | Arg |  |  |
| A02.063 | walleye dermal sarcoma virus retropepsin | 23 |  |  | Asn/Gln |  |  |  |  |  |
| A26.001 | omptin | 53 |  |  |  | **basic** |  |  |  |  |
| A32.002 | RC1339 g.p. ({Rickettsia conorii}) | 771 | Arg/Lys | Arg/Lys | Arg/Lys |  |  |  |  |  |
| C01.009 | cathepsin V | 1626 |  |  | Gly |  |  |  |  |  |
| C01.013 | cathepsin X | 43 |  |  |  |  |  |  |  |  |
| C01.024 | endopeptidase-B ({Hordeum}-type) | 63 |  |  |  | Pro |  |  |  |  |
| C01.033 | cathepsin L1 ({Fasciola} sp.) | 195 | Trp |  | Gln/Ile | Cys/Ile | Ile |  | Ile/Met |  |
| C01.034 | cathepsin S | 3067 |  |  | Asp |  |  |  |  |  |
| C01.036 | cathepsin K | 2105 |  |  | Asp |  |  |  |  |  |
| C01.040 | cathepsin H | 41 | acidic |  |  |  | Cys/His |  |  |  |
| C01.046 | falcipain-2 | 148 | Ile | Ile |  | Cys/Ile | Ile | Cys/Gln | Ile | Ile |
| C01.057 | vinckepain-2 | 23 |  |  |  |  |  |  |  | Glu/"-" |
| C01.060 | cathepsin B | 620 |  |  |  |  |  |  | Met |  |
| C01.063 | falcipain-3 | 125 | Ile |  | Cys/Ile | Ile/Met | Ile |  | Ile |  |
| C01.070 | rhodesain | 32 |  | acidic |  |  |  |  |  |  |
| C01.075 | cruzipain | 57 | aromatic |  |  |  |  |  |  |  |
| C01.086 | aminopeptidase C | 22 |  |  |  |  |  |  | NAP/"-" |  |
| C01.101 | cathepsin B-like peptidase, nematode | 42 |  |  | Gln/Pro |  | Ile/Met |  |  |  |
| C01.129 | cathepsin L3 ({Fasciola} sp.) | 72 |  |  |  |  |  | Met/Trp | Phe/Trp | His/Met |
| C01.130 | CSCP3 peptidase ({Clonorchis}-type) | 56 |  |  |  |  |  |  | Ile/Trp |  |
| C01.142 | cathepsin L2 ({Fasciola} sp.) | 54 |  |  |  | Cys/Thr | Cys/Trp |  |  |  |
| C02.002 | calpain-2 | 380 |  |  |  |  |  |  | Cys |  |
| C05.001 | adenain | 24 |  |  | Gly/Ser |  |  |  |  |  |
| C12.003 | ubiquitinyl hydrolase-L3 | 21 |  |  |  | Gly |  |  |  |  |
| C13.004 | legumain, animal-type | 2222 |  |  |  | Trp | Cys |  |  |  |
| C14.001 | caspase-1 | 171 | Arg | Trp | Trp |  | Pro/Trp | Trp | His | Trp/Tyr |
| C14.003 | caspase-3 | 580 |  |  |  | **Asp**/Glu |  |  |  |  |
| C14.004 | caspase-7 | 466 |  |  |  | **acidic** |  | Cys |  |  |
| C14.005 | caspase-6 | 1289 |  | Trp |  | **Asp** |  |  |  |  |
| C14.006 | caspase-2 | 289 |  |  | Arg/Lys | **Asp** | **small** |  |  | Trp |
| C14.009 | caspase-8 | 67 |  |  | Trp | **Asp** |  | Trp | Trp |  |
| C25.001 | gingipain RgpA | 25 |  |  |  | **Arg** |  |  |  |  |
| C25.002 | gingipain K | 43 | acidic |  |  | Arg/**Lys** |  |  |  |  |
| C30.001 | coronavirus picornain 3C-like peptidase-1 | 32 |  |  | **aliphatic** | **Gln** | **small** |  |  |  |
| C30.005 | SARS coronavirus picornain 3C-like peptidase | 24 |  |  |  | Gln/Gly |  |  |  |  |
| C48.002 | SENP1 peptidase | 23 | **Gln** | **Thr**/Tyr | **Gly** | **Gly** |  |  |  |  |
| G01.002 | aspergilloglutamic peptidase | 67 |  | Trp | Gly/Ile |  | His/Trp | Cys/Met |  | Pro |
| M01.001 | aminopeptidase N | 38 |  |  |  |  |  |  |  |  |
| M01.002 | lysyl aminopeptidase (bacteria) | 21 |  |  |  |  |  |  |  |  |
| M01.010 | cytosol alanyl aminopeptidase | 44 |  |  |  |  |  |  |  |  |
| M01.018 | endoplasmic reticulum aminopeptidase 1 | 52 |  |  |  |  |  |  |  |  |
| M01.024 | ERAP2 aminopeptidase | 22 |  |  |  |  |  |  |  |  |
| M03.002 | neurolysin | 118 | acidic |  |  |  |  |  |  |  |
| M03.011 | tropolysin | 31 |  |  | **small** |  |  |  |  |  |
| M04.001 | thermolysin | 3968 |  |  |  |  | Arg/Cys |  |  |  |
| M04.014 | bacillolysin | 40 | aromatic | acidic |  |  |  |  |  |  |
| M09.002 | collagenase G/A ({Clostridium histolyticum}) | 106 |  |  |  |  |  |  | Asn/Lys | Trp |
| M09.003 | bacterial collagenase H | 155 | Phe |  |  |  | **small** | Arg/Trp | Lys/Met | Trp |
| M09.005 | collagenase ColT ({Clostridium tetani}) | 172 | Ile |  | Tyr | Arg/Lys |  | Arg/Trp | Trp | Arg |
| M10.001 | matrix metallopeptidase-1 | 75 |  |  |  | Ile/Trp |  | Cys | Phe/Trp |  |
| M10.002 | matrix metallopeptidase-8 | 105 |  |  | Cys |  |  | Cys | Met/Phe |  |
| M10.004 | matrix metallopeptidase-9 | 357 | Trp | Trp | Trp |  | Cys |  |  |  |
| M10.008 | matrix metallopeptidase-7 | 186 | Trp | Cys/Trp | Trp | Trp |  | Cys |  | His/Trp |
| M10.009 | matrix metallopeptidase-12 | 185 | Trp |  | Cys | Cys/Trp |  |  | Met | Trp |
| M10.013 | matrix metallopeptidase-13 | 144 |  |  | Asp/Cys | Trp | Cys/Pro | Cys |  | Trp |
| M10.014 | membrane-type matrix metallopeptidase-1 | 129 | Trp/Tyr |  | Trp | Cys/Val | Cys/His | Cys | Trp | Cys/Met |
| M10.024 | membrane-type matrix metallopeptidase-6 | 46 |  |  |  |  |  |  | Met/Trp |  |
| M12.001 | astacin | 208 |  |  |  | Cys |  | Arg/Lys |  |  |
| XM12.001 | meprin A complex peptidase | 70 | Ile/Met | Cys | Ile |  | Asn/Pro | Asn/Ile |  | Asn/Ile |
| M12.032 | LAST peptidase ({Limulus}-type) | 77 | Trp/Tyr | Cys |  |  |  |  |  | Cys/Met |
| M12.138 | jararhagin | 37 | Phe/Tyr |  |  |  |  |  |  |  |
| M12.140 | bothropasin | 62 | Arg/Trp |  |  |  |  |  |  |  |
| M12.217 | ADAM17 peptidase | 56 |  |  |  |  |  |  |  | Cys/Lys |
| M12.221 | ADAMTS4 peptidase | 57 |  |  | Cys |  |  | Met/Trp |  |  |
| M13.001 | neprilysin | 57 | Ile/Thr |  |  | Ile/Thr |  |  | Asn |  |
| M14.005 | carboxypeptidase E | 22 |  |  |  |  | **basic** |  |  |  |
| M14.017 | carboxypeptidase A4 | 58 | Cys/Trp |  |  |  |  |  |  |  |
| M14.018 | carboxypeptidase A6 | 37 |  |  |  |  |  |  |  |  |
| M14.023 | CPG70 carboxypeptidase ({Porphyromonas gingivalis}) | 22 |  |  |  |  | Arg/Lys |  |  |  |
| M16.002 | insulysin | 63 |  |  | Trp |  |  |  | Met/Trp | Gln/Trp |
| M16.009 | eupitrilysin | 46 | Glu/Trp |  |  |  |  |  |  |  |
| M24.001 | methionyl aminopeptidase 1 ({Escherichia}-type) | 929 |  |  |  | **Met** | His/Trp |  |  |  |
| M24.002 | methionyl aminopeptidase 2 | 128 |  |  |  | **Met** |  | Cys | Cys |  |
| M24.026 | aminopeptidase P3 | 46 |  |  |  |  |  |  |  |  |
| M24.028 | mitochondrial methionyl aminopeptidase | 84 |  |  |  | **Met** |  |  | Cys/Met | Met |
| M24.038 | aminopeptidase P ({Plasmodium falciparum}-type) | 22 |  |  |  |  | **Pro** |  |  |  |
| M28.002 | aminopeptidase Ap1 | 65 |  |  |  |  | Pro | Ile/Tyr | Ile |  |
| M28.014 | carboxypeptidase Q | 33 |  |  |  |  |  |  |  |  |
| M35.004 | peptidyl-Lys metallopeptidase | 1912 |  |  |  |  | **Lys** |  |  |  |
| M42.004 | PhTET2 aminopeptidase | 23 |  |  |  |  |  |  |  |  |
| M49.001 | dipeptidyl-peptidase III | 24 |  |  |  |  |  |  |  |  |
| M61.001 | glycyl aminopeptidase | 26 |  |  |  |  |  |  |  |  |
| M72.001 | peptidyl-Asp metallopeptidase | 21 |  |  |  |  | **Asp**/Cys |  |  |  |
| M9E.004 | membrane Pro-Xaa carboxypeptidase | 20 |  |  |  |  |  |  |  |  |
| P01.001 | DmpA aminopeptidase | 33 | Ala/"-" |  |  |  |  |  |  |  |
| S01.001 | chymotrypsin A (cattle-type) | 790 |  |  |  | Gly/Ser |  |  |  |  |
| S01.005 | mast cell peptidase 4 ({Rattus}) | 40 |  |  |  | Phe/Tyr |  |  |  |  |
| S01.015 | tryptase beta | 59 |  |  |  | Arg/Lys |  |  |  |  |
| S01.021 | DESC1 peptidase | 109 | acidic | Asp |  | **Arg** | Pro |  |  |  |
| S01.029 | kallikrein-related peptidase 14 | 70 |  |  |  |  |  |  | Cys/Met |  |
| S01.047 | human airway trypsin-like peptidase |  | acidic |  |  | **Arg** |  |  |  |  |
| S01.072 | matriptase-3 | 113 | His/Trp |  | His/Trp |  |  |  |  |  |
| S01.081 | kallikrein-related peptidase 15 | 1357 |  |  |  | **Arg**/Lys |  |  |  |  |
| S01.131 | elastase-2 | 368 |  |  |  | His/Trp |  |  |  |  |
| S01.133 | cathepsin G | 252 | Lys |  |  |  |  |  |  |  |
| S01.134 | myeloblastin | 50 | acidic |  |  |  |  | Met |  |  |
| S01.135 | granzyme A | 303 |  | Trp |  | **basic** |  |  | Trp | Arg/Trp |
| S01.136 | granzyme B, rodent-type | 360 | Arg |  | Arg/Cys | **acidic** | Arg/Pro | Trp |  |  |
| S01.139 | granzyme M | 748 |  |  |  | Trp |  | Trp |  | Trp |
| S01.140 | chymase ({Homo sapiens}-type) | 104 | His/Tyr | Tyr |  | **aromatic** |  |  | Cys/Trp |  |
| S01.143 | tryptase alpha | 23 |  |  |  | Arg/Lys |  |  |  |  |
| S01.146 | granzyme K | 20 |  |  |  | Arg/Lys |  |  |  |  |
| S01.151 | trypsin 1 | 17157 |  |  |  | Arg/Lys |  |  |  |  |
| S01.160 | kallikrein 1 | 31 |  |  |  |  | acidic |  |  |  |
| S01.161 | kallikrein-related peptidase 2 | 65 |  |  |  | **basic** |  | Cys/Tyr |  |  |
| S01.162 | kallikrein-related peptidase 3 | 72 | aromatic |  |  |  | Pro/Trp |  |  |  |
| S01.174 | mesotrypsin | 20 |  |  |  | Arg/Lys |  |  |  |  |
| S01.212 | plasma kallikrein | 37 |  |  |  | Arg/Lys |  |  |  |  |
| S01.216 | coagulation factor Xa | 56 |  |  |  | Arg/Lys |  |  |  |  |
| S01.217 | thrombin | 163 | Asp/Cys |  |  | **basic** |  | Asp/Trp | Cys/Trp | Met |
| S01.224 | hepsin | 109 |  |  |  | **Arg** |  | Cys/Trp | Arg/Trp |  |
| S01.233 | plasmin | 126 | Trp | Trp |  | **basic** | Pro/Trp | Met/Trp |  | Trp |
| S01.236 | kallikrein-related peptidase 4 | 80 | Cys/Met |  |  |  |  |  | Cys |  |
| S01.244 | kallikrein-related peptidase 8 | 24 |  |  |  | Arg/Lys |  |  |  |  |
| S01.251 | kallikrein-related peptidase 4 | 116 |  | Cys | Cys |  |  |  |  |  |
| S01.267 | streptogrisin E | 23 |  |  |  | Ala/**Glu** |  |  |  | Asp/"-" |
| S01.269 | glutamyl endopeptidase I | 3922 |  |  |  | **Glu** |  |  |  |  |
| S01.271 | glutamyl peptidase BL | 72 |  |  |  | Asp/**Glu** |  |  | Trp |  |
| S01.278 | HtrA2 peptidase | 70 |  | Trp |  |  |  |  |  |  |
| S01.280 | lysyl endopeptidase (bacteria) | 785 |  |  |  | **Lys** |  |  |  |  |
| S01.302 | matriptase | 115 | acidic | Asp/Trp |  | **Arg** |  |  |  |  |
| S01.306 | kallikrein-related peptidase 13 | 20 |  |  |  | **basic** |  |  |  |  |
| S01.308 | matriptase-2 | 157 | Cys |  | Trp | **Arg**/Lys |  | Asp/Trp | Cys/Trp | Cys/Trp |
| S01.312 | SplE peptidase ({Staphylococcus aureus}) | 27 |  |  |  |  |  |  |  |  |
| S01.458 | chymase 1 ({Mus musculus}-type) | 44 |  |  |  | **aromatic** |  |  |  |  |
| S07.001 | flavivirin | 21 |  |  | **basic** | **Arg**/Lys |  |  |  |  |
| S08.019 | lactocepin I | 102 | Cys | Cys | Cys/Trp | Cys/Trp | Cys/Trp | Cys | Cys | Cys/Trp |
| S08.070 | kexin | 184 | Cys/Trp | Pro | **basic** | **Arg**/Lys |  |  |  |  |
| S08.071 | furin | 188 |  | Cys/Tyr |  | **Arg** | Ile | Cys/Tyr |  | Trp |
| S08.072 | PCSK1 peptidase | 78 |  |  |  | **Arg**/Lys |  | Met/Trp |  | Cys/Val |
| S08.073 | PCSK2 peptidase | 184 | Asp/Cys | Cys/Trp |  | **Arg** |  | His |  |  |
| S08.074 | PCSK4 peptidase | 99 |  |  |  | **Arg**/Lys |  |  |  |  |
| S08.075 | PCSK6 peptidase | 100 |  |  |  | **Arg**/Lys |  |  |  | Trp/Tyr |
| S08.076 | PCSK5 peptidase | 122 |  |  |  | **Arg**/Lys | Trp |  |  | Trp |
| S08.077 | PCSK7 peptidase | 108 |  |  |  | **Arg**/Lys |  |  | Met/Trp | Trp/Tyr |
| S08.109 | KPC2-type peptidase | 110 | Cys/Thr | Cys/Trp |  | **basic** |  |  |  | Cys |
| S08.112 | ARA12 peptidase | 50 |  |  |  |  |  |  |  | Asp/**Tyr** |
| S08.116 | lactocepin-3 | 158 | Cys | Cys | Cys | Cys | Cys | Cys |  | Cys |
| S09.003 | dipeptidyl-peptidase IV (eukaryote) | 27 |  |  |  |  |  |  |  |  |
| S09.010 | oligopeptidase B | 31 |  | acidic |  | **basic** |  |  |  |  |
| S10.006 | Mername-AA083 peptidase | 70 | Cys/Ile | Ile | His/Ile |  | Cys/His |  |  |  |
| S10.014 | carboxypeptidase O | 88 | His/Ile | His | His/Ile | Cys | Cys/His |  |  |  |
| S26.001 | signal peptidase I | 320 | Trp |  | Pro | **Ala** | Pro | Arg |  | Cys |
| S26.008 | thylakoidal processing peptidase | 51 |  |  |  | **small** |  |  |  |  |
| S26.010 | signalase (animal) 21 kDa component | 335 | Trp | Trp/Tyr |  | His/Met | Pro | Met |  |  |
| S26.016 | SpsB signal peptidase | 42 |  |  |  | **Ala** |  |  |  |  |
| XS26.001 | signal peptidase complex (animal) | 1677 |  |  |  | His |  |  |  |  |
| S28.002 | dipeptidyl-peptidase II | 25 |  |  |  |  |  |  |  |  |
| S46.001 | dipeptidyl-peptidase 7 ({Porphyromonas gingivalis}-type) | 21 |  |  |  |  |  |  |  |  |
| S50.004 | blotched snakehead birnavirus Vp4 peptidase | 30 |  |  |  | Aba/Ala |  |  |  |  |
| S53.002 | sedolisin-B | 44 | **Pro** |  |  | **Phe** | Nph |  |  |  |
| S53.005 | kumamolisin-B | 56 | **Pro** |  |  | **Phe** | **Phe** |  |  |  |
| S54.004 | AarA peptidase | 46 | **Ile** |  |  | **Ala** |  |  | **Gly** | **Ser** |
| S80.001 | prohead peptidase gp175 (Pseudomonas aeruginosa phage phiKZ) | 25 |  | **small** |  | **Glu** |  |  |  |  |
| XT01.002 | 26S proteasome peptidase complex (eukaryote) | 139 | Asp | His/Met |  |  |  |  |  | His |
| XT01.003 | 20 S immunoproteasome peptidase complex (eukaryote) | 226 | Cys | Cys | Cys | Cys |  | Cys/Trp |  | Cys |
| U74.001 | neprosin | 109 |  | Cys/Trp | His/Phe |  |  |  |  | Trp |
